# Sinus tympani revisited for planning retrofacial approach—radiologic study in pneumatized temporal bones and its surgical implications

**DOI:** 10.1007/s00405-022-07576-4

**Published:** 2022-08-05

**Authors:** Tomasz Wojciechowski, Robert Bartoszewicz, Kazimierz Szopiński

**Affiliations:** 1 Department of Descriptive and Clinical Anatomy, The Medical University of Warsaw, 5 Chalubinskiego St., 02004 Warsaw, Poland; 2 Department of Otorhinolaryngology, Head and Neck Surgery, The Medical University of Warsaw, 1a Banacha St., 02097 Warsaw, Poland; 3 Department of Dental and Maxillofacial Radiology, The Medical University of Warsaw, 6 Bienieckiego St., 02097 Warsaw, Poland

**Keywords:** 3D segmentation, Computed tomography, Retrofacial approach, Stapedius muscle, Sinus tympani, tympanic sinus, Temporal bone, Pneumatization

## Abstract

**Background and purpose:**

Retrofacial approach (RFA) is an access route to sinus tympani (ST) and it is used in cholesteatoma surgery, especially when type C ST is encountered. It may also be used to gain an access to stapedius muscle to assess the evoked stapedius reflex threshold. The primary object of this study was to evaluate the morphology of sinus tympani and its relationship to facial nerve (FN) and posterior semicircular canal (PSC) in context of planning retrofacial approach in pneumatized temporal bones.

**Methods:**

CBCT of 130 adults were reviewed. The type of sinus tympani was assessed according to Marchioni's classification. Width of entrance to sinus tympani (STW), depth of ST (STD), distance between the posterior semicircular canal and facial nerve (F-PSC), distance between the latter plane to the floor of ST at the right angle (P-ST) were measured at level of round window (RW) and pyramidal ridge (PR).

**Results:**

All of the bones were well-aerated and classified in Dexian Tan pneumatization group 3 or 4. Type B of ST is dominant (70.8%) in adult population with no history of inflammatory otologic diseases, followed by type C (22.7%) and then type A (6.5%). The depth of ST (STD) presented significant deviations (ANOVA, *p* < 0.05) among all three types. STW reaches greater values on the level of PR. F-PSC does not correlate with type of ST. In over 75% of examined type C sinus tympani the distance P-ST was less than 1 mm.

**Conclusions:**

The qualitative classification of the sinus tympani into types A, B and C, introduced by Marchioni is justified by statistically significant differences of depth between individual types of tympanic sinuses. The STW distance reaches greater values inferiorly—it may suggest that RFA should be performed in infero-superior manner rather than opposite direction. Preoperative assessment of temporal bones CT scans gives very important information about size of sinus tympani and distance between FN and PSC.

**Supplementary Information:**

The online version contains supplementary material available at 10.1007/s00405-022-07576-4.

## Introduction

Sinus tympani (ST), a recess of retrotympanum located between the mastoid part of facial nerve and the posterior semicircular canal, has been a subject of many anatomical, otologic and radiologic investigations [1, 2]. The difficulty of surgical access to this area when clearing the middle ear in cases of cholesteatoma is notorious [3]. Lately, researchers reported that the prevalence of a very deep ST (type C) varies between 4% in diseased ears [4] to as much as 34% in normal, well-aerated temporal bones [5]. The intraoperative visualization of a deep ST is often inadequate, especially when a posterior extension is encountered [3, 4]. To provide control over cholesteatoma removal, indirect microtympanoscopy of the ST may be performed with the assistance of a Zini mirror inserted through the external acoustic meatus [6]. Alternatively, endoscopic approaches can guarantee a very detailed visualization and exploration of a deep ST [4].

**Fig. 1:**
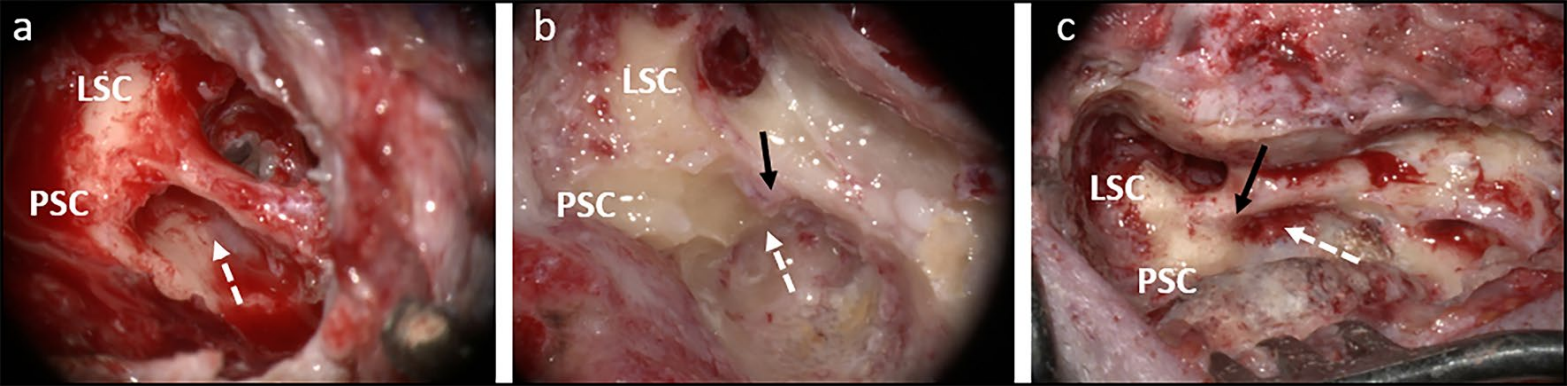
**a** Right ear; retrofacial approach in child during cochlear implantation. **b, c** Right ears; retrofacial approach in different cases of canal wall up tympanoplasties for chronic otitis media; *LSC* lateral semicircular canal, *PSC* posterior semicircular canal, black arrow—stapedius muscle belly, white arrow—corridor for retrofacial approach

Retrofacial approach (RFA) is a posterior access route to ST, used to explore the posterior-most spaces of the medial retrotympanum, hypotympanum and infralabyrinthine area (Fig. 1) [1, 2, 7]. Until recently, it has been used mainly as an alternative to the canal wall down procedure giving an opportunity to spare the posterior wall of external acoustic meatus during removal of inflammatory tissue from the middle ear [1, 3]. There is an increasing number of reports using RFA to access the area of the round window and promontory in difficult cochlear implantations [8], especially in cases of congenital middle ear malformations [9]. Arnold et al. [10] described RFA as a safe access to stapedius muscle to assess the evoked stapedius reflex threshold. At the level of the round window, the sinus tympani is narrowed by the stapedius muscle chamber; however, its width and depth increase inferiorly [11, 12]. Bearing that in mind, there is a need to assess the dimensions of ST on different levels to increase safety of RFA given the proximity of the facial nerve and posterior semicircular canal.

The aim of the study was to analyze the ST morphology in context of Marchioni’s classification in adults with well-aerated, pneumatized temporal bones and to check whether preoperative analysis of distances between structures such as the facial nerve and posterior semicircular canal may help in planning retrofacial approach.

## Materials and methods

In this retrospective study anonymized sets of cone-beam computed tomography (CBCT) studies of the temporal bones performed in the Department of Dental and Maxillofacial Radiology, Medical University of Warsaw, between February 2016 and June 2017 were analyzed. All studies of the facial cranium were performed as a part of standard work-up of patients with dental or maxillofacial indications. The scans were performed with a Planmeca Promax 3D Mid CBCT scanner (Planmeca USA, INC, Roselle, Illinois, USA), source voltage 90 kV, current 12 mA, voxel dimensions 400 × 400 × 400 µm. The exclusion criteria were: radiologic signs of trauma or post-traumatic changes in the skull base, previous otologic surgery (canal wall up tympanoplasty, canal wall down tympanoplasty or other), radiologic signs of inflammation (e. g. soft tissue or mucus in air cells), tumors or anomalies of external, middle and internal ear. Temporal bones were also evaluated in terms of pneumatization. Two scales were used—proposed by Virapongse et al. [13] and Dexian Tan et al. [14]. Virapongse et al. divided temporal bones into three types—sclerotic (score: 0), diploic (1) and pneumatized (2, 3 and 4). Dexian Tan et al. proposed 4 groups—hypopneumatization (1), moderate (2), good (3) or hyperpneumatization (4). The only included temporal bones were pneumatized—according to Virapongse score of 2, 3 and 4 and Dexian Tan groups 3 and 4. A group of 130 sets of images (59 male and 71 female, 260 temporal bones) was enrolled in the study as the final group. The age of the patients involved in the analysis ranged from 12 to 84 (mean age 38 ± 16 years).

In the next phase, the scans were analyzed with the RadiAnt DICOM Viewer 2020.2 (64-bit; Medixant, Poznan, Poland). Window level was set to 400 Hounsfield Units (HU) and window width to 4000 HU. All measurements were performed twice by an experienced anatomist using the described methods in everyday practice (TW).

An additional microCT scan of an anatomic specimen gathered from the Department of Clinical and Descriptive Anatomy of the temporal bone was performed. The scan was obtained with the scanner Phoenix Xray (GE Sensing & Inspection Technologies, Wunstorf, Germany) with parameters: voxel dimensions 0.07 × 0.07 × 0.07 mm; the exposition performed with source voltage of 120 kV and current of 70 mA.. The data was processed with a Mimics Innovation Suite 24.0 (Materialise, Belgium) software to obtain 3D images for better understanding of surgical conditions expected. Is has been added to this paper as a PDF supplementary material for anyone who wants to analyze an example of retrotympanic area in context of the results presented in this paper.

Obtained data were analyzed statistically with StatSoft Statistica 13.3 and IBM SPSS Statistics software. The Shapiro–Wilk test was used to verify the normality of distribution. For each of measured parameters descriptive statistics were calculated: mean value ± standard deviation (SD), min–max range. Subsequently, the dependence of all variables on side and sex of patients was determined with ANOVA test. The obtained results were tested with Student’s *t* test for parametric variables, and Mann–Whitney *U*-test for nonparametric variables. The differences between the qualitative variables were assessed with the chi-squared test. Relationships between continuous variables were assessed using the Pearson's r correlation coefficient or Spearman's rho correlation coefficient. The *p* < 0.05 was considered significant.

### Standardization procedure related to the method of measurements

To fully visualize the complexity of the topographic relations between the structures of petrous bone, an analysis of standard sections obtained in the horizontal, sagittal and frontal planes is often not sufficient.

Therefore, in each case of the present study, additional multiplanar reformation (MPR) planes were obtained to assure perfect symmetry of the images. The additional planes included a tilted horizontal plane showing a "signet" image of the lateral semicircular canal with vestibule, which is a standard procedure when interpreting images of the temporal bones. The images were also assessed in the Pöschl plane [15], i.e., showing the longitudinal plane of the anterior semicircular canal. Finally, images in the plane of the posterior semicircular canals were obtained and assessed. In this way, cross sections of all semicircular canals and other structures of the bony labyrinth, as well as typical images of the structures inside the tympanic cavity are obtained [16].

### Measurements related to sinus tympani and feasibility of retrofacial approach

**Fig. 2:**
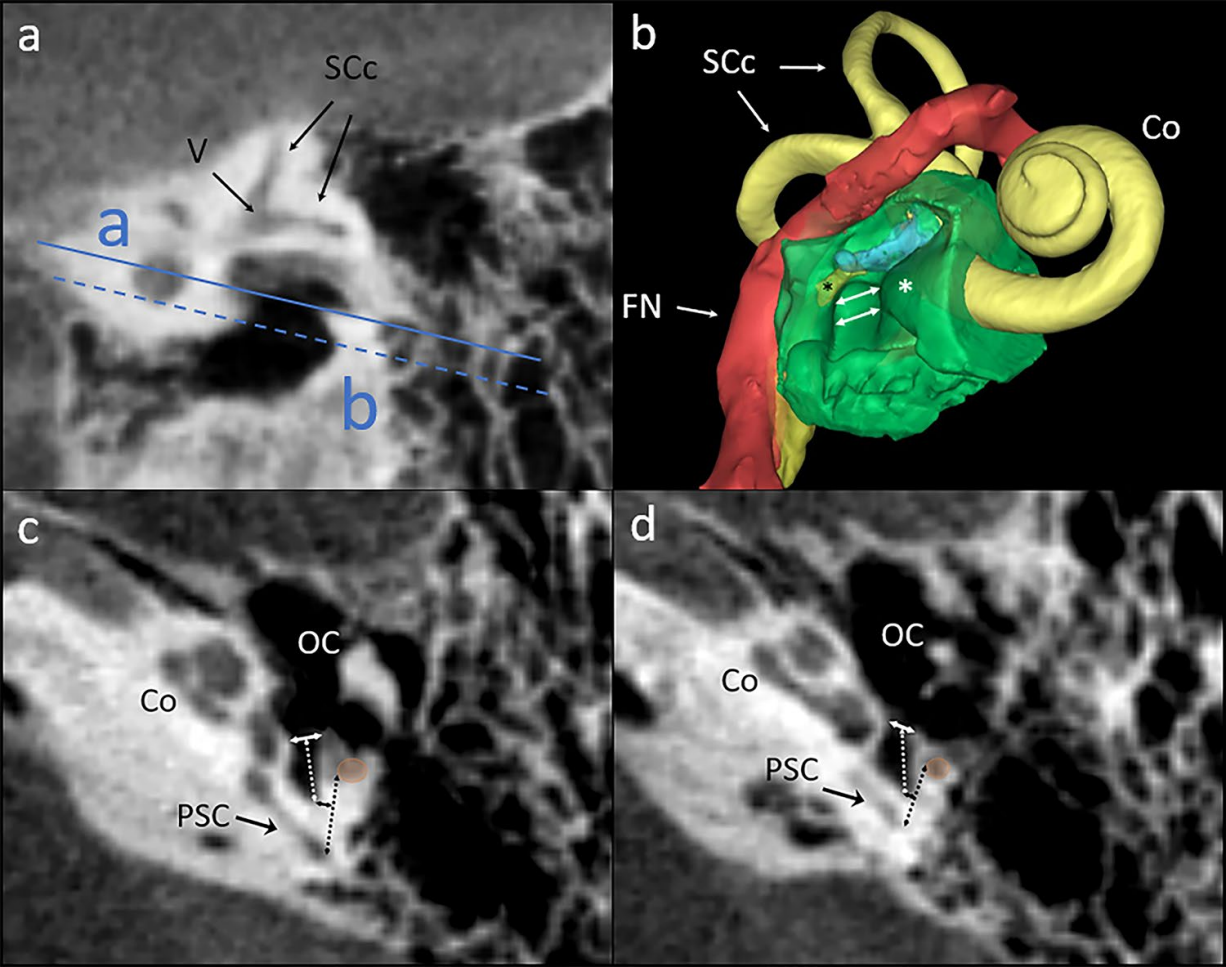
**a** Sagittal cross section of temporal bone showing the levels of measurements taken (a—round window (RW) level, b—pyramidal ridge (PR) level); **b** 3D reconstruction of the area of measurements, white lines between asterisks indicate STW at RW level (upper) and PR level (lower);** c** measurements at RW level;** d** measurements at PR level; *v* vestibule, *SCc* semicircular canals *Co* cochlea, black asterisk stapedius muscle emerging from pyramidal eminence, white asterisk—promontory, *FN* facial nerve, *OC* ossicular chain, *PSC* posterior semicircular canal, orange circle—facial nerve, white solid line—sinus tympani width (STW), white dotted line—sinus tympani depth (STD), black dotted line—F-PSC distance, solid black line—P-ST distance

The measurements were performed on two levels (Fig. 2):

(a) at the level of the promontory and round window (RW) and pyramidal eminence,

(b) at the level of the pyramidal ridge (PR) and lower portion of the tympanic sinus.

The following distances were measured:

- width of the entrance to the sinus tympani (STW),
- depth of the sinus tympani (STD),
- distance between the posterior semicircular canal and facial nerve (F-PSC),
- distance between the line linking the posterior semicircular canal and facial nerve latter and the floor of ST (P-ST).

**Fig. 3:**
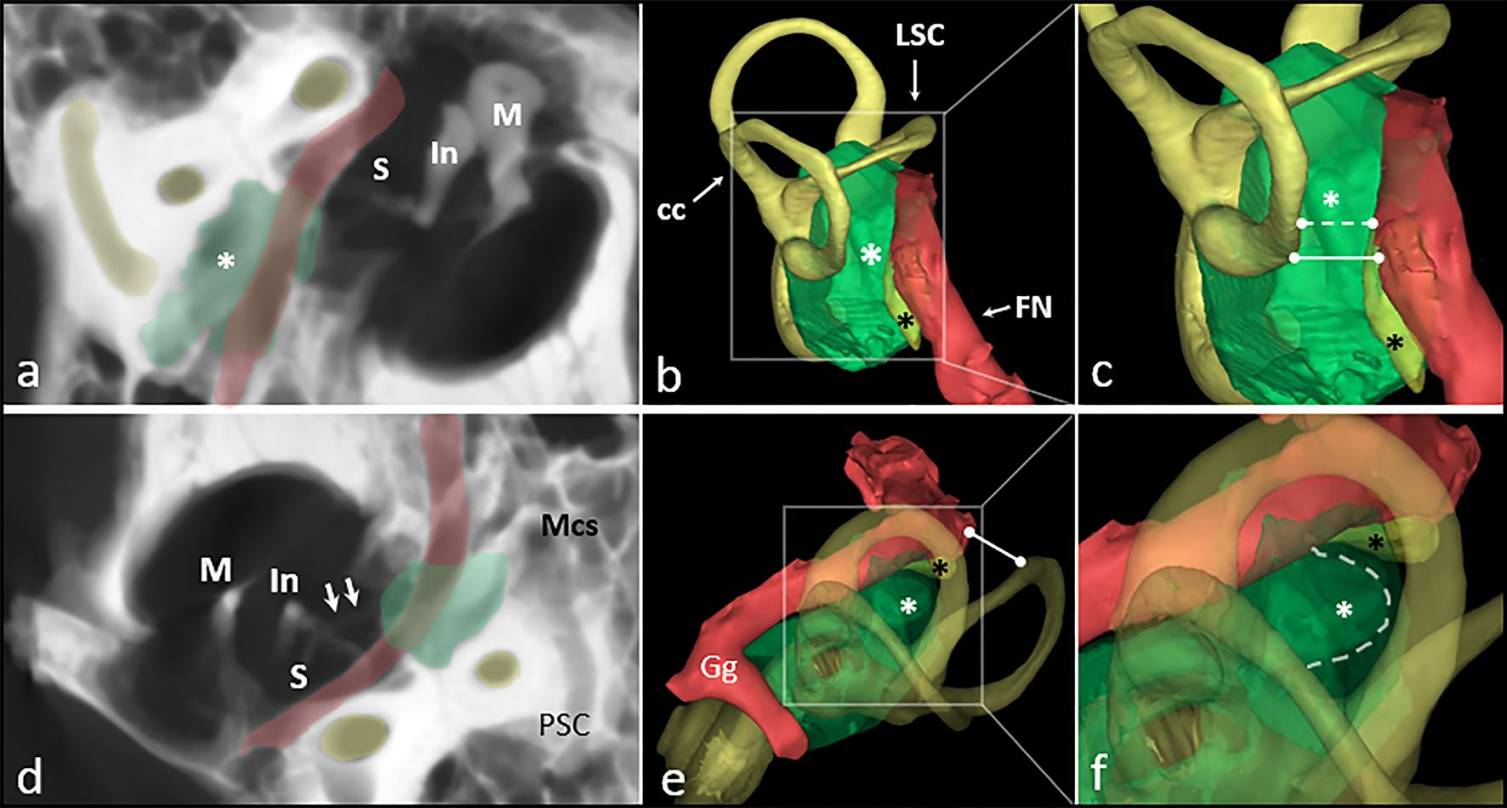
**a** Average intensity projection of temporal bone microCT, posterior view; green area—sinus tympani, red area—facial nerve, yellow area—semicircular canals, white asterisk—sinus tympani, *M* malleus, *I* incus, *S* stapes;** b** General view of 3D reconstruction of RFA area; green area—sinus tympani with bone around, red area—facial nerve, yellow area—labyrinth, white asterisk—sinus tympani, *cc* common crus, *LSC* lateral semicircular canal, *FN* facial nerve, black asterisk—stapedius muscle; **c** detailed view of 3D reconstruction of RFA area**;** white dotted line—F-PSC measurement at higher level (RW), white line—F-PSC measurement at lower level (PR); **d** average intensity projection of temporal bone microCT, superior view; green area—sinus tympani, red area—facial nerve, yellow area—semicircular canals, *M* malleus, *I* incus, *S* stapes; white arrows—stapedius muscle tendon, *Mcs* mastoid cells system, *PSC* posterior semicircular canal; **e** general view of 3D reconstruction of ST area**;** green area—sinus tympani with bone around, red area—facial nerve, yellow area—labyrinth, white asterisk—sinus tympani, black asterisk—stapedius muscle belly, *Gg* geniculate ganglion; **f** detailed view of 3D reconstruction of ST area; white dotted line—posterior border of sinus tympani between facial nerve and posterior semicircular canal;

The measured distances are defined in Table 1 and shown in Figs. 2 and 3. This method has been previously used and acknowledged in assessment of ST dimensions in children [5, 17].

**Table 1:** Variables and symbols used in the study and a description of how they were defined

Parameter	Description
STW	Distance between the most lateral point of promontory and the tip of pyramidal eminence/pyramidal ridge
STD	Distance between the center of STW and the most posterior point of ST
F-PSC	distance between the most lateral point of the posterior semicircular canal and the most medial point of the mastoid part of facial canal
P-ST	distance between the F-PSC line and the most posterior point of the ST

**Fig. 4:**
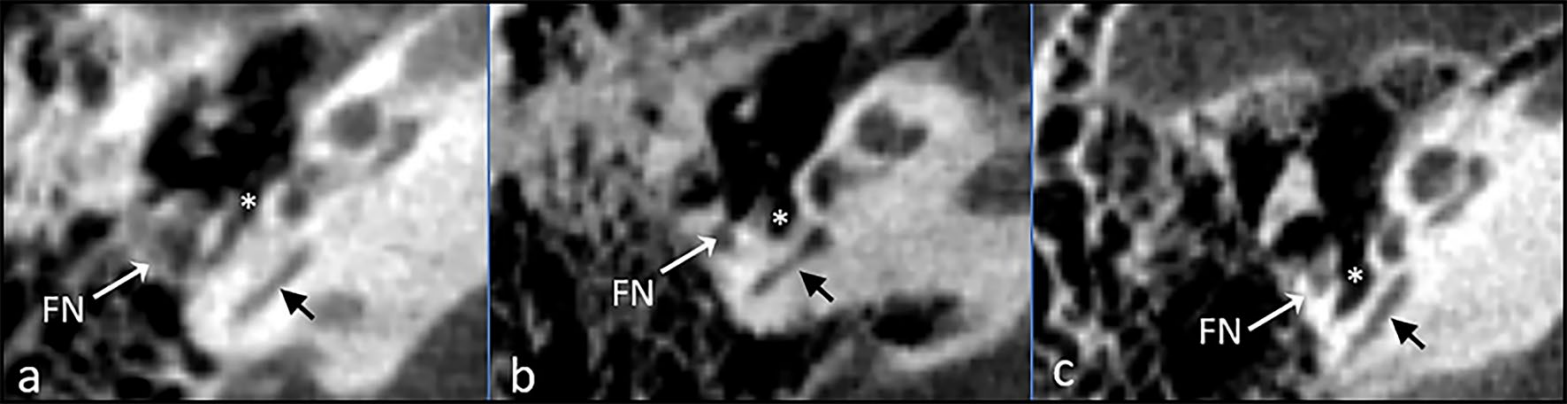
Different types of sinus tympani; **a** type A sinus tympani; **b** type B sinus tympani; **c** type C sinus tympani; *FN* facial nerve, black arrow—posterior semicircular canal, white asterisk—sinus tympani

In the next step, after ST was identified and measured, the type of ST was assessed, according to modified Marchioni’s classification [4, 5]. Type A is a shallow ST, that does not reach the mastoid segment of the facial nerve. Type B is a ST reaching the mastoid segment of the facial nerve, but without extension behind that level. Type C is a deep ST with postero-medial or postero-lateral extension in relation to the mastoid segment of the facial nerve (Fig. 4).

This study has been approved by the Ethics Committee of Medical University of Warsaw (decision number AKBE/185/2019), and abides by the 1964 Helsinki Declaration and its later amendments or comparable ethical standards. The AQUA Checklist was followed to ensure the clarity of this anatomical report.

## Results

From 260 temporal bones assessed in terms of pneumatization, there were 210 bones assigned to Dexian Tan group 4 and 50 bones—to group number 3. According to Virapongse classification all of the bones were well-pneumatized. Bilaterally, Dexian Tan group 4 was found in 101 sets and group 3 in 21 sets of images. In 8 sets there were temporal bones group 3 on one side and group 4 on the other.

The sinus tympani was found and assessed on all evaluated scans. In 260 analyzed temporal bones, type B of the sinus tympani, found in 70.8% of temporal bone was the most common. Type C was found in 22.7% temporal bones cases, and type A in 6,5% of temporal bones. The were no significant differences in numbers of particular ST types between males and females (chi-squared test, *p* = 0.594) as well as between left and right sides (chi-squared test, *p* = 0.072). The number of particular types of sinus tympani on the right and left side, in males and females is presented in Table 2.

**Table 2:** Number of identified types of ST according to side and sex

	Type A	Type B	Type C	Total
Side				
Left	9 (6.9%)	84 (64.6%)	37 (28.5%)	130 (50%)
Right	8 (6.2%)	100 (76.9%)	22 (16.9%)	130 (50%)
Sex				
Females	11 (7.7%)	101 (71.1%)	30 (21.1%)	142 (54.6%)
Males	6 (5.1%)	83 (70.3%)	29 (24.6%)	118 (45.4%)
Total	17 (6.5%)	184 (70.8%)	59 (22.7%)	260 (100%)

The dimensions of the sinus tympani at the level of the round window and at the level of the pyramidal ridge were not significantly correlated with each other (round window: *r* = − 0.11, *p* = 0.068; pyramidal ridge: *r* = 0.03, *p* = 0.614). The width of the entrance to the sinus tympani (STW) at the level of the pyramidal ridge is usually larger than at the level of the round window (*p* = 0.002). On the left side significantly larger STW are observed (regardless of gender and measurement level) than on the right side (*p* < 0.001). The depth of the sinus tympani (STD) is significantly greater at the level of the pyramidal ridge (p = 0.017). Significant sex differences were also noticed—regardless of the side and level, a greater STD was noted in men than in women (*p* = 0.009). Significant correlation between the width of the entrance to the sinus tympani (STW) at the RW level and the PR level was observed (right side *r* = 0.24, *p* = 0.005; left side *r* = 0.25, *p* = 0.005). Similar relationships were shown for STD. The correlation was positive and significant on both sides (right side: *r* = 0.69, *p* < 0.001; left side: *r* = 0.64, *p* < 0.001).

Mean values, standard deviations, and min–max ranges for all measured parameters with respect to types A, B and C on different measurement levels are presented in Table 3.

**Table 3:** Mean values, SD and min–max range of the measured parameters according to type of ST on different levels of measurements in order—mean ± SD (range) [mm]

Level of measurements	Round window (RW)			Pyramidal ridge (PR)		
ST Type	A	B	C	A	B	C
STW	1.98 ± 0.67 (1.06–3.51)	1.77 ± 0.40 (0.96–3.21)	1.72 ± 0.35 (0.87–2.73)	2.04 ± 0.49 (1.34–3.21)	1.86 ± 0.48 (0.90–3.81)	1.88 ± 0.53 (0.99–3.70)
STD	2.23 ± 0.88 (1.04–4.20)	3.19 ± 0.68 (0.90–5.18)	3.85 ± 0.78 (2.02–5.49)	2.34 ± 0.85 (0.99–3.74)	3.24 ± 0.62 (1.51–5.07)	4.14 ± 0.72 (2.82–5.66)
F-PSC	4.19 ± 0.90 (1.81–5.45)	4.42 ± 0.88 (1.42–6.71)	4.68 ± 0.67 (2.77–6.52)	3.99 ± 0.89 (2.10–5.65)	3.91 ± 0.73 (1.19–6.34)	4.24 ± 0.74 (1.90–5.85)
P-ST	2.18 ± 0.68 (1.10–3.71)	1.66 ± 0.66 (0.00–5.63)	0.93 ± 0.50 (0.00–2.55)	1.90 ± 0.80 (0.83–4.03)	1.53 ± 0.66 (0.47–4.40)	0.75 ± 0.48 (0.00–2.20)

In females, the width of the entrance to the sinus tympani (STW) at the level of the pyramidal ridge on left side correlated with F-PSC distance (*r* = − 0.35, *p* = 0.003). In addition, in females, the width of the entrance to the sinus tympani (STW) at the level of round window on the right side correlated with the F-PSC distance (*r* = 0.29, *p* = 0.015).

**Fig. 5:**
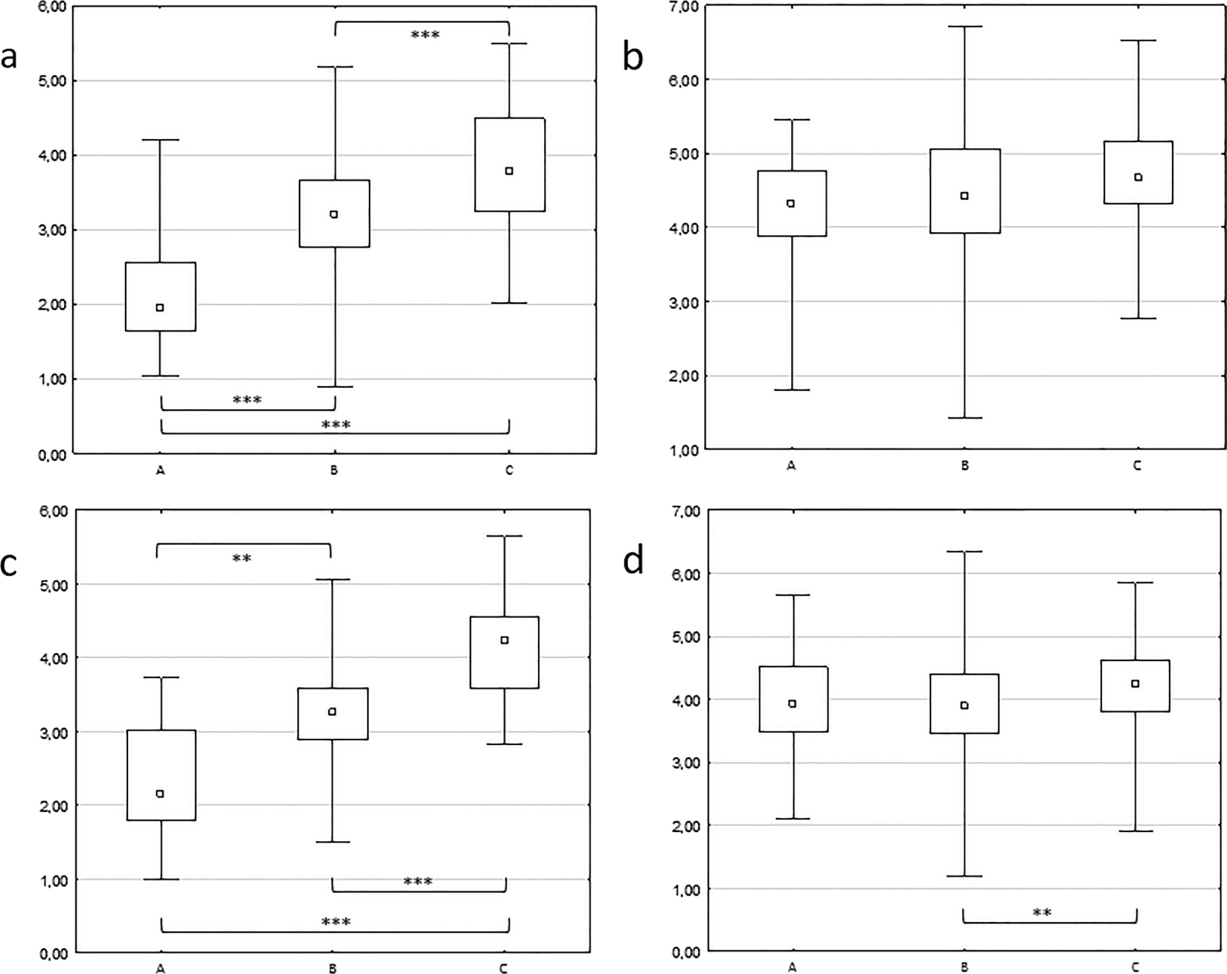
**a** Median depth (STD) of different types of sinus tympani at RW level; **b** median value of F-PSC in different types of sinus tympani at RW level; **c** median depth of the sinus tympani (STD) in different types of sinus tympani at PR level; **d** median value of F-PSC distane in different types of sinus tympani at PR level; **p ≤ 0.01 ***p ≤ 0.001

The relationships between the type of ST (A, B and C) and its dimensions (STW, STD, F-PSC, P-ST) were analyzed (Table 4). The depth of ST (STD) presented significant deviations among all the three types (Fig. 5) on both levels. However, this does not allow for the determination of reference values for individual ST type, as the ranges of the value distribution overlapped largely. This means that the type of tympanic sinus cannot be determined by numeric values. The results confirm, however, that the bottom of a deeper ST (type C) is very close to the line drawn between the facial nerve and posterior semicircular canal (P-ST). In over 75% of examined type C sinus tympani the distance P-ST was less than 1 mm.

**Table 4:** Kruskal–Wallis ANOVA for the dimensions of the ST depending on its types (general population, *n* = 260)

	*p*	Multiple comparisons
RW level		
STW	0,494	**–-**
STD	< 0,001	A < B < C***
F-PSC	0,042	**–-**
P-ST	< 0,001	A > B* > C***
PR level		
STW	0,269	**–**
STD	< 0,001	A < B ** < C***
F-PSC	0,018	B < C**
P-ST	< 0,001	A, B > C***

*p* *p* value

*p ≤ 0.05 **p ≤ 0.01 ***p ≤ 0.001

## Discussion

Almost 200 years ago Meckel [18] discovered a small pouch localized medially to the pyramidal eminence, starting thus the research which continues into present days. In 1889, Steinbrugge found that ST may extend behind and under the facial canal rendering impossible a complete cleansing from diseased tissues [19]. Cheatle stated that it is often impossible to clean thoroughly the tympanic sinus without endangering the facial nerve [20]. Only the second half of the twentieth century brought more detailed information about morphology of the sinus tympani, and attempts were made to explore this space. The first measurements of the ST were made by Dworacek [21], and then Farrior [22] drew attention to the difficult access to the ST when performing posterior tympanotomy. Donaldson et al. [23] analyzed temporal bone specimens harvested from children and adults to demonstrate constant occurrence of ST and its highly variable depth. They conclude that tissues affected by chronic inflammation can be removed even from deep tympanic sinuses, but this requires "altering of the approach", i.e., using the retrofacial approach. Subdivision of the *retrotympanum* into four sinuses was proposed by Proctor [24] and followed by Smyth with his team [25]. Pickett et al. [1] analyzed STs in computed tomography, writing about the "luxury" that aural surgeons have at their disposal by planning operations based on images obtained with this method. Further researchers have demonstrated one bone specimens the possibility of accessing the deep portion of the ST through the mastoid cells [17, 26].

The anatomic description of ST, although studied by many authors, has only recently been unified due to development of middle ear surgery techniques, especially endoscopic ones. The sinus tympani, being the most constant retrotympanic recess has variable depth in different individuals. Marchioni et al. [4] proposed a qualitative classification taking into account the spatial relations of the sinus and the mastoid segment of the facial nerve, distinguishing types A, B and C (Fig. 3). However, they did not discuss neither the absolute depth nor differences between types of STs that may be encountered. Interestingly, even though depth (STD) of ST is higher in men than in woman, it does not correlate with differences in numbers of particular types of STs. The cause of this finding is that the ranges of STD of STs type B and C overlap as the type depends on the position related to the facial nerve. One may encounter ST type B deeper than ST type C in terms of absolute depth. This finding may support the qualitative categorization into types A, B and C rather than quantitative (depth in milimeters). In several studies other authors report that the type B is dominant, but there are differences in occurrence between types A and C [27–29]. Type C is second common type of ST in well-pneumatized temporal bones (Table 5). Recent study on children suggest that temporal bones not affected by chronic inflammatory diseases may develop a larger pneumatic system [5], including a ST deeper than in patients with history of chronic otitis media [27]. Lately, Hool et al. found that ST type A may be of higher prevalence in cholesteatoma patients. That is why one has to remember that impaired pneumatization of the mastoid may be associated with a less pneumatized retrotympanum [30]. As our group of temporal bones was pneumatized, well-aerated only, we wish that the measurements done are to compared with further studies, where diploic and sclerotic mastoids are included. One has to bare in mind that though the term mastoid pneumatization is often used interchangeably with mastoid aeration iit does not have to always represent the same. If mastoid air cells are filled with water-density material (e.g., mucus, soft tissue), one may call that region pneumatized but not aerated. It is because the pneumatization process has already been finished but cells may not be aerated at the very moment of image acquisition and interpretation.

**Table 5:** Comparison of the percentage distribution of individual types of ST in the literature and the analyzed material

ST Type	Marchioni et al. [4]	Baklaci et al. [27]		El-Anwar et al. [28]	Takahashi et al. [29]	Wojciechowski and Skadorwa [5]	Analyzed material
	Whole group (*n* = 296) (%)	Whole group (*n* = 202) (%)	Adult pneumatized mastoids (*n* = 53) (%)	Adult pneumatized mastoids (*n* = 200) (%)	Whole group (*n* = 31)	Ongoing pneumatization (*n* = 300) (%)	Adult pneumatized mastoids (*n* = 260) (%)
A	33.1	25.2	7.5	28	29	6.3	6.5
B	62.5	69.8	77.4	71	71	59.3	70.8
C	4.4	5	15.1	1	0	34.3	22.7

The exploration of ST is necessary not only in cases of otitis media with cholesteatoma or granulation tissues. The ST may also be invaded by tympanic and jugulo-tympanic paraganglioma or endolymphatic sac tumours [31]. In such situations RFA may be a part of wider operation including petrosectomy with facial nerve re-routing [32]. One has to remember that a high-riding jugular bulb may be a significant obstacle in RFA performed as a first step to access hypotympanum and infralabyrinthine area rather than as a route to ST only [33].

Arnold et al. report that RFA can be also used to access the area of stapedius muscle to assess evoked stapedius reflex thresholds after cochlear implantation [10]. Volk et al. suggest that every patient that is a candidate for RFA should be evaluated with use of 3D reconstruction of the area based on the CT scans obtained preoperatively [12]. Both authors are aware of potential risks—damaging the posterior semicircular canal and stapedius muscle. It may seem that the experience gained by drilling for blue line of anterior semicircular canal during middle fossa approach for vestibular schwannoma resection or vestibular neurectomy may be of use when practicing RFA [34]. When a surgeon wants to prevent an accidental damage, the main objective is to find posterior semicircular canal and to expose its blue line [12]. The belly of stapedius muscle is enclosed by bony tissue narrowing ST at the level of round window [16, 35]. Our study suggest that STD and F-PSC enlarges inferiorly. This information may be of use for otologic surgeon to plan an inferior approach from the area of hypotympanum. It may also decrease the risk of damaging the belly of stapedius muscle [2]. The use of CBCT has been acknowledged in literature when it comes to visualize preoperative conditions in an individual temporal bone [36]. We suggest the posterior semicircular canal and facial nerve have to be evaluated before every RFA to the area of ST. 3D reconstruction may also help to easier understand the complicated relations between these structures [12, 37]. We believe that the ability to do a proper analysis of multiplanar reconstructions has to be in the armamentarium of every otologic surgeon. In authors’ opinion a preoperative CT scan is obligatory in cases of disease invading medial spaces of the retrotympanum, in re-operations, and when suspicion of middle ear malformation is raised.

Chen et al. [17] conducted study in which they recommended access to the ST via RFA in cases when the distance between the facial nerve and posterior semicircular canal is greater than 3 mm. In our material this condition is met by as many as 235 (90%) bones at the RW level and 229 (88%) bones at the PR level. Chen et al. [17], such as previous authors [1, 3], state that RFA is contraindicated in presence of anomalies of the course of the mastoid segment of facial nerve. Such situations are very rare and their occurrence most often coexists with middle ear malformations [9, 38]. Other authors point that RFA may be helpful during cochlear implantation, especially when RW cannot be visualized properly by posterior tympanotomy [8]. The high-riding jugular bulb may also make RFA more demanding for a surgeon [33]. An important phenomenon that has not been previously described in the literature, are statistically significant differences in the distance between the facial nerve and the posterior semicircular canal (F-PSC) in bones with STs type B and C. This means that wider RFA can be performed in bones with ST type C. Moreover, it turned out that the potential width of RFA in material analyzed in this study reaches higher values ​​than reported by other authors [2, 17]. Our study also suggest that the width of the entrance to the ST is similar or even smaller than the distance between the PSC and facial canal. This is another argument in favor of performing RFA in clinical situations that require it. The dimensions of entrance to the ST are similar to those reported in other studies and mean value of these measurements is smaller than 2 mm (Table 6), while the mean F-PSC distance is larger than 4 mm. The F-PSC distance measured in adults is comparable to the distance in children what may suggest that it does not change throughout life [5].

**Table 6:** Comparison of the distribution of values of ST dimensions in the literature and the analyzed material

	Sample size (*n*)	ST type	STW	STD	F-PSC
Ozturan et al. [2]	327	*nn*	1.59 ± 0.14	2.34 ± 0.17	3.11 ± 0.10
Chen et al. [17]	24	*nn*	1.98 ± 0.71	2.25 ± 0.91	3.67 ± 0.58
El-Anwar et al. [28]	200	A	1.82 ± 0.78	2.52 ± 0.5	*nn*
		B			
		C			
Wojciechowski and Skadorwa [5]	300	A	1.51 ± 0.49	1.93 ± 0.41	4.66 ± 0.99
		B	1.48 ± 0.41	3.07 ± 0.77	4.93 ± 0.76
		C	1.46 ± 0.35	4.19 ± 0.96	4.91 ± 0.66
Analyzed material	260	A	1.98 ± 0.67	2.23 ± 0.88	4.19 ± 0.90
		B	1.77 ± 0.40	3.19 ± 0.68	4.42 ± 0.88
		C	1.72 ± 0.35	3.85 ± 0.78	4.68 ± 0.67

The P-ST dimension in type C sinus tympani in 75% is less than 1 mm—it is comparable or even smaller than the width of petrosquamous septum or bone protecting the lateral semicircular canal (eminence of LSC) [40, 41]. The surgical implication of such phenomenon is vital—such a bone may be pierced with a blunt probe rather than burr and removed with a House curette. Increase of the F-PSC distance and STW in the lower portion of the sinus tympani should raise a question concerning the development of this structure. Is the sinus tympani a single outpouching of mesotympanum? Or maybe the superior part of the sinus tympani is formed along with cochlea, footplate of stapes and stapedius muscle, and the inferior part of the sinus tympani derives from the ossification center of the styloid eminence and its size depends on the pneumatization process of the hypotympanum and mastoid air cell system? The two above mentioned grooves merge and form the sinus. In this model it may be compared to formation of a greater palatine canal—when a vertical groove on the palatine bone becomes a canal closed by the maxilla.

At last, it is necessary to compare the potential width of posterior tympanotomy with width of entrance to ST from mesotympanum and width of RFA (F-PSC distance). Several authors suggest that chorda tympani can be sacrificed to extend posterior tympanotomy, leaving facial nerve the only vital structure to be protected from unintended damage [39]. Su et al. [39] pointed that extended posterior tympanotomy width is 4.01 ± 0.56 mm, also Adad et al. [42] reported comparable results—3.78 ± 0.87 mm. It is also similar to our measurements of the F-PSC distance. The results suggest that accessing the ST should be done by means of posterior tympanotomy or RFA rather than through narrow entrance from mesotympanum, especially when the ST is deep. Of course, RFA potentially puts at risk the posterior semicircular canal, but only when accessing the ST from above [10]. On the other hand, the inferior approach inferior puts the jugular bulb in danger [33]. To detect the presence of a high-riding jugular bulb, a preoperative CT scan is mandatory. It may also provide information about the potential width of accesses, so the surgeon can plan ahead what set of tools is necessary, including the diameter of burrs.

### Limitations of the study

The main limitation of this retrospective study was lack of access to the medical histories of the patients, especially in terms of ear diseases. The patients did not undergo any otosurgical interventions and it is difficult to compare the gathered population with other authors in terms of pre- and post-operative conditions. The group was limited to the patients with well-pneumatized temporal bones and that gives a perspective of normal anatomy rather than in terms of pathology. In addition, quantitative depth of the sinus tympani was arbitrarily defined as the distance between the center of STW and the poster point of ST, not the clinically useful depth posterior to the anterior edge of the facial nerve (Marchioni's C).

The main delimitations of the study are neither intra-observer nor inter-observer data. That is why we were able only to compare our results to other found in the literature of the subject.

### Further research destinations

The large majority of adults with cholesteatoma have sclerotic, non-pneumatized or minimally pneumatized temporal bones. That is why further research has to be oriented to compare the regional pneumatization of retrotympanum and whole temporal bones in group of patients with cholesteatoma.

## Conclusions

1. The qualitative classification of the sinus tympani into types A, B and C, introduced by Marchioni et al. [4] is justified by statistically significant differences of depth between individual types of tympanic sinuses.
2. Type B of ST is dominant in adult population. Type C is second dominant in well-pneumatized temporal bones. The P-ST distance is less than 1 mm. in more than 75% of cases of type C sinus tympani
3. The STD reaches greater values at the inferior level of measurements (pyramidal ridge) than at the superior level (pyramidal eminence and round window) It may suggest that RFA should be performed in infero-superior manner rather than opposite direction when one wants to quickly enter the sinus tympani.
4. Preoperative assessment of temporal bones CT scans gives very important information about size of sinus tympani and distance between FC and PSC. It may alter an approach to eradication of disease from medial spaces of retrotympanum. It may also increase the safety of approaches in difficult cochlear implantations, and make easier the planning of intraoperative evoked stapedius reflex threshold measurements.

## Supplementary Information

Below is the link to the electronic supplementary material.

Supplementary file1 (PDF 2799 KB)

## Data Availability

Please contact authors for data requests (Tomasz Wojciechowski MD PhD—email: tomasz.wojciechowski@wum.edu.pl).
